# Exosomes From Human Umbilical Cord Mesenchymal Stem Cells Treat Corneal Injury via Autophagy Activation

**DOI:** 10.3389/fbioe.2022.879192

**Published:** 2022-04-11

**Authors:** Shisi Ma, Jiayang Yin, Lili Hao, Xiao Liu, Qi Shi, Yuyao Diao, Guocheng Yu, Lian Liu, Jiansu Chen, Jingxiang Zhong

**Affiliations:** ^1^ Department of Ophthalmology, The First Affiliated Hospital of Jinan University, Jinan University, Guangzhou, China; ^2^ Institute of Ophthalmology, Medical College, Jinan University, Guangzhou, China; ^3^ The Sixth Affiliated Hospital of Jinan University, Jinan University, Dongguan, China

**Keywords:** autophagy, apoptosis, corneal injury, exosome, inflammation, ocular surface regeneration

## Abstract

Corneal injury (CI) affects corneal integrity and transparency, deteriorating the patient’s quality of life. This study aimed to explore the molecular mechanisms by which exosomes secreted from human umbilical cord mesenchymal stem cells (hucMSC-Exos) affect autophagy in human corneal epithelial cells (HCECs) and CI models. We isolated and identified hucMSC-Exos using nanoparticle tracking analysis, transmission electron microscopy, and western blotting. The effects of hucMSC-Exos combined with autophagy regulators on HCECs and CI mice were assessed using cell viability assays, scratch assay, cell cycle assay, apoptosis assay, corneal fluorescein staining, haze grades, pathological examinations, western blotting, and quantitative polymerase chain reaction (qPCR). *In vitro* results indicated that hucMSC-Exos combined with the autophagy activator had positive effects in promoting the cell proliferation, migration capacity, and the cell cycle by upregulating the proportions of cells in the S phase and the expression of PCNA, Cyclin A, Cyclin E, and CDK2. Meanwhile, the combination treatment reduced the apoptotic rate of HCECs. *In vivo* results indicated that hucMSC-Exos especially combined them with the autophagy activator significantly alleviated corneal epithelial defects and stromal opacity, reduced the levels of the apoptotic markers Bax and cleaved Caspase-3, reduced the inflammatory response products TNF-α, IL-1β, IL-6, and CXCL-2, and increased the Bcl-2. This was achieved by upregulating pAMPK/AMPK and pULK1/ULK1 ratios, and Beclin-1 and LC3B II/I, and by downregulating the pmTOR/mTOR ratio and p62. In contrast, clinical indications, apoptosis, and inflammation were aggravated after the application of the autophagy inhibitor. HucMSC-Exos combined with an autophagy activator significantly enhanced HCECs functions and alleviated corneal defects, apoptosis, and inflammation by activating the autophagy signaling pathway, AMPK-mTOR-ULK1, providing a new biological therapy for corneal wound healing and ocular surface regeneration.

## Introduction

The cornea is situated on the most anterior segment of the eye and acts as the first refractive element (Stern et al., 2018). Corneal injury (CI) affecting the epithelium and stroma may damage the transparency and integrity of the cornea, leading to corneal neovascularization, conjunctivalization, scarring, or even complete blindness (Kacham et al., 2021). Steady and rapid regeneration of the corneal epithelium and stroma are vital for preventing pathogenic invasion of the endothelium; however, this remains a clinical challenge (Kokado et al., 2018). In addition to traditional medical treatment and corneal transplantation, current developments in treatment have focused on exosomes, cell transplantation, biopolymers, and other approaches (Mobaraki et al., 2019; Muijzer et al., 2019).

Therapies involving mesenchymal stem cells (MSCs) obtained from adipose, bone marrow, or umbilical cord tissues promoted ocular surface healing (Reinshagen et al., 2011; Cejkova et al., 2013; Holan and Javorkova, 2013; Rohaina et al., 2014; Kacham et al., 2021). With the advantages of high self-renewing capacity, low immunogenicity, and fewer ethical issues than other stem cells, human umbilical cord MSCs (hucMSCs) are better suited to clinical application (Weiss et al., 2008). However, stem cell applications face several challenges, including uncertain differentiation, immune incompatibility, infection, mass production and stable storage (Su et al., 2021). As cell-free therapies, exosomes, ranging from 30 to 150 nm in size, are an efficient component of intercellular communication that may solve the problem of MSCs as biological drug carriers (Azmi et al., 2013; Becker et al., 2016; Théry et al., 2018a; Tang et al., 2021). Exosomes derived from MSCs exerted therapeutic effects on ocular surface diseases, such as dry eye diseases (Kapsogeorgou et al., 2005; Li et al., 2016; Wang G. et al., 2021), corneal wounds (Han et al., 2017; Samaeekia et al., 2018; Li N. et al., 2019; Shojaati et al., 2019), and corneal defects (Coulson-Thomas et al., 2013; Wang et al., 2020b).

Multivesicular body-related secretory pathways and autophagy are interconnected at many levels (Raudenska et al., 2021). On the one hand, extracellular vesicles carry cargos to induce target cells autophagy by activating the associated signaling pathways. On the other hand, exosome/amphisome biogenesis and degradation are greatly influenced by autophagy machinery (Xu et al., 2018).

In all eukaryotic cells, autophagy is a self-degradation process that maintains homeostasis in the synthesis, degradation, and circulation of cellular components (Morgan-Bathke et al., 2013). Autophagy is known to positively influences dry eye (Seo et al., 2014; Byun et al., 2017; Ma et al., 2019; Liu et al., 2020a; Liu et al., 2020b; Lyu et al., 2020; Wang B. et al., 2021), keratitis (Choi et al., 2013; Li et al., 2020; Han et al., 2021), keratoconus (Iqbal et al., 2013), and corneal impairment (Wang Y. et al., 2020; Li et al., 2021), suggesting that it might have potential for treating ocular surface diseases. Beclin-1, microtubule-associated protein one light chain three beta (LC3B), and sequestosome 1 (SQSTM1/p62) are marker proteins involved in autophagic processes. Beclin-1 is generally known as an essential constituent in the initiation of autophagy (Schmitz et al., 2016). The LC3-phosphatidylethanolamine conjugate (LC3-II), formed by cytoplasmic LC3 (LC3-I), aggregates on the surface of autophagosomes during autophagy (He et al., 2015). P62 functions as a cargo protein that interacts with ubiquitin; it is degraded upon fusion with lysosomes, and reflects the level of autophagic flux (Boyle and Randow, 2013). Adenosine 5′-monophosphate-activated protein kinase (AMPK) is a vital molecular marker in the autophagic upstream pathway (Yang et al., 2020), and its phosphorylation induces autophagy by activating Unc-51 like kinase 1 (ULK1)-Beclin1 and inhibiting the mammalian target of rapamycin (mTOR) (Kim et al., 2011). Rapamycin is a serine/threonine kinase that specifically inhibits mTOR, and plays a crucial role in regulating cell growth and metabolism, especially in the autophagic pathway (Shah et al., 2017). Dorsomorphin/Compound C is widely regarded as an AMPK inhibitor that inhibits autophagy by phosphorylating mTOR (Seo et al., 2016; Chen et al., 2020). Autophagy could be directly linked to exosomal biogenesis through associated molecular mechanisms or organelles in cases of retinal detachment (Ma et al., 2020) and age-related macular degeneration (Wang et al., 2009; Atienzar-Aroca et al., 2018).

To the best of our knowledge, it isunknown whether the exosomes secreted from hucMSCs (hucMSC-Exos) and associated autophagy can exert a therapeutic effect in events of CI. The purpose of this research is to observe the mechanisms through which hucMSC-Exos and autophagy affect CI. For this, we performed cell viability assays, scratch wound assay,cell apoptosis assay, cell cycle assay *in vitro*, corneal fluorescein staining, haze grades, pathological staining, terminal-deoxynucleoitidyl transferase mediated nick end labeling (TUNEL) assay, western blotting, and quantitative polymerase chain reaction *in vivo* Furthermore, we studied the occurrence and development of autophagy in CI models.

## Materials and Methods

### Cell Culture and Validation of hucMSCs

The isolation of hucMSCs was approved by the Medical Ethical Committee of the First Affiliated Hospital of Jinan University (Approval No. KY-2021-067). The donors provided written informed consent in accordance with the Declaration of Helsinki. Cells were isolated and cultured as described by Gu et al. (Gu et al., 2020). Osteogenic, adipogenic, and chondrogenic differentiation (Gibco, United States) experiments were assessed through Alizarin Red, Oil Red-O, and Safranine O (Sigma Aldrich, United States) stainings as described by the manufacturer, and images were observed under a microscope (ICC50, Leica, Germany). The hucMSCs were incubated with the marker antibodies CD73 (550,257, BD Pharmingen, United States), CD90 (559,869, BD Pharmingen), CD105 (561,443, BD Pharmingen), CD34 (555,824, BD Pharmingen), CD45 (555,482, BD Pharmingen) and HLA-DR (560,651, BD Pharmingen) for 30 min, centrifuged at 300 × *g* for 5 min, and analyzed by flow cytometry (BD, FACS Canto II). Human corneal epithelial cells transformed with Simian virus 40 (HCECs) were cultivated in Dulbecco’s Modified Eagle Medium/F-12 (DMEM/F12, Gibco) supplemented with 10% fetal bovine serum (FBS; Gibco), 10 ng/ml recombinant human epidermal growth factor (E9644, Sigma Aldrich), 5 μg/ml insulin (I2643, Sigma Aldrich), and 1% penicillin-streptomycin (Gibco). Cells were maintained at 37 C in a 5% CO_2_ incubator (Forma 3, Thermo Scientific, United States) until they reached 80–90% confluence.

### Isolation, Identification and Quantification of hucMSC-Exos

Check the composition of DMEM/F12 medium before use. Serum-free conditioned medium DMEM/F12 (11320, Gibco) with hucMSCs was collected and centrifuged at 300 × *g* for 10 min, 2,000 × *g* for 10 min, and 15,000 × *g* for 30 min. The supernatant was filtered, concentrated, and ultracentrifuged at 120,000 × *g* for 70 min (Optima L-80X, Beckman Coulter, United States). Exosomes were collected and resuspended in phosphate buffer solution (PBS; Gibco). Their size, polydispersity, morphology, and surface marker antibodies CD9 (ab263019, Abcam, UK), CD63 (ab134045, Abcam), CD81 (ab109201, Abcam), TSG101 (ab181606, Abcam), HSP70 (ab125011, Abcam) and Calnexin (ab133615, Abcam) were determined by nanoparticle tracking analysis (NTA; NS300, Malvern, UK), transmission electron microscopy (TEM; Talos Arctic G2, Thermo Scientific, United States), and western blotting. As previously reported, the number of exosomes was quantified by the EXOCET exosomes quantification kit (EXOCET96A-1, System Biosciences, United States), according to the manufacturer’s instructions (Samaeekia et al., 2018; Soares Martins et al., 2018). The colorimetric assay measuring the absorbance at 405 nm of acetylcholinesterase enriched activity was performed along with the standard curve for quantification.

### Exosome Fusion Assay

The hucMSC-Exos were added into a mixture of PKH26 and Diluent C (MINI26, Sigma Aldrich) for 5 min. After adding 0.5% FBS to terminate the reaction, exosomes were ultracentrifuged in the dark at 120,000 × *g* for 90 min. HCECs and mouse corneas were treated with labelled hucMSC-Exos or PBS for 24 h, after which nuclei were stained with 4’,6-diamidino-2phenylindole (DAPI; Leagene, China), and photographed using a confocal microscope (LSM880, Carl Zeiss, Germany).

### Preparation of Autophagy Regulators and Group Design

**FIGURE 1:**
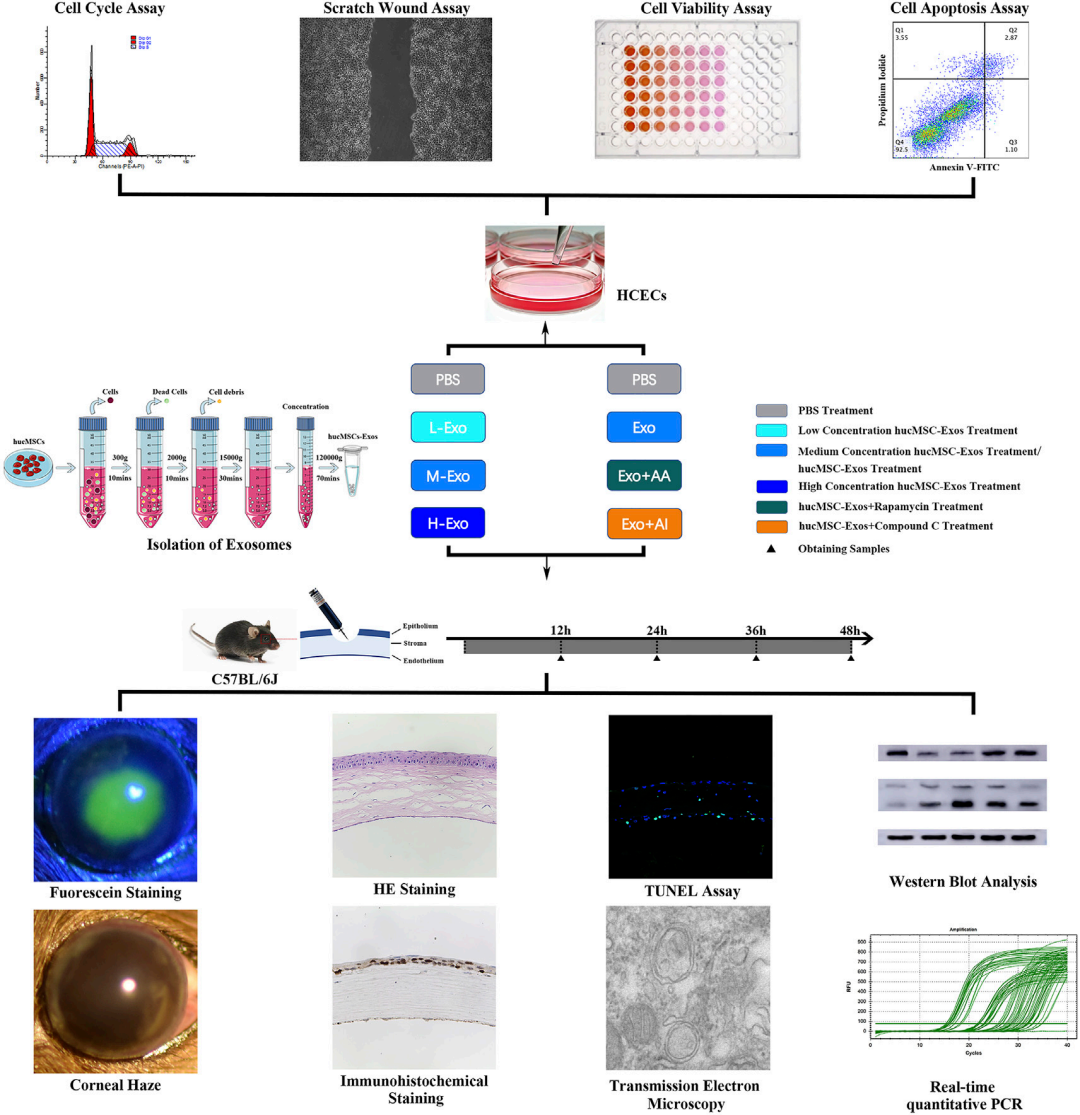
Schematic protocol. Schematic representation of the isolation of the exosomes secreted from hucMSCs, *in vitro* experiments of HCECs, and *in vivo* experiments of corneal injury mouse.

The autophagy activator (AA) Rapamycin (V900930, Sigma Aldrich) and the autophagy inhibitor (AI) dorsomorphin/Compound C (P5499, Sigma Aldrich) were dissolved and stored at 4 C. Group designs and schematic protocol of *in vitro* and *in vivo* experiments are shown as follows (Figure 1).

HCECs in the control group were treated with PBS; HCECs in the Exo group were treated with 1 × 10^6^/μl hucMSC-Exos; HCECs in the AA group were treated with 50 nM Rapamycin; HCECs in the AI group were treated with 5 μM Compound C; HCECs in the Exo + AA group were treated with 1 × 10^6^/μl hucMSC-Exos and 50 nM Rapamycin; and HCECs in the Exo + AI group were treated with 1 × 10^6^/μl hucMSC-Exos and 5 μM Compound C.

Normal corneas in the control group were treated with PBS; injured corneas in the CI + PBS group were treated with PBS; injured corneas in the CI + L-Exo group were treated with 1 × 10^5^/μl hucMSC-Exos; injured corneas in the CI + M-Exo group were treated with 1 × 10^6^/μl hucMSC-Exos; injured corneas in the CI + H-Exo group were treated with 1 × 10^7^/μl hucMSC-Exos; injured corneas in the CI + Exo + AA group were treated with 1 × 10^6^/μl hucMSC-Exos and 10 μM Rapamycin; and injured corneas in the CI + Exo + AI group were treated with 1 × 10^6^/μl hucMSC-Exos and 50 μM Compound C.

### Cell Proliferation Assay

HCECs were seeded into 96-well plates in triplicates and grown to 60% confluence. The cells were cultured in growth factor-starved medium with various treatments. The medium was replaced with 10 µl of Cell Counting Kit-8 (CCK-8; Beyotime, China) solution after 24 h and incubated for 1 h at 37 C. Optical density was measured at 450 nm using a microplate reader (Varioskan LUX, Thermo Scientific).

### Cell Migration Assay

Fixed with a sterile ruler, confluent HCECs were scratched with a sterile standard 10 μl pipette tip (T-300-R-S, Axygen, United States), then treated for 24 h. Wound closure was recorded using a fluorescent microscope (DMI8, Leica) and measured using ImageJ software (1.52, NIH, United States). The wound closure ratio = [Cell-free area (0 h)- Cell-free area (24 h)]/Cell-free area (0 h) × 100 % (Fang et al., 2016).

### Cell Apoptosis Assay

HCECs were mixed with 2.5 μl FITC Annexin V and with 2.5 μl propidium iodide staining solution (556547, BD) and incubated in darkness for 15 min. Pellets were resuspended in binding buffer and apoptosis rates were analyzed using flow cytometry.

### Cell Cycle Assay

Harvested HCECs were incubated with cold 70% ethanol for 2 h at -20°C. The cells were washed with PBS to remove the ethanol then resuspended in PI/RNase Staining Buffer (550825, BD) for 15 min and immediately analyzed using a flow cytometer (Gallios, Beckman Coulter).

### Corneal Debridement and Treatment

The animal procedures were approved by the Laboratory Animal Ethics Committee of Jinan University (Approval No. IACUC-20200925-02). Experiments were conducted in accordance with the ARVO Statement for the Use of Animals in Ophthalmic and Vision Research. Male C57BL/6 J mice aged 6–8 weeks (Beijing Vital River Laboratory Animal Technology Co. Ltd., China) were anaesthetized using pentobarbital sodium solution (70 mg/kg) and proparacaine hydrochloride eye drops (Santen, Japan). The whole corneal epithelium and anterior stroma were scratched with AlgerBrush II corneal remover (Alger, United States) after being marked with 2.5 mm trephine. Each group was treated three times daily.

### Evaluation of Corneal Fluorescein Staining Scores and Haze Grades

Fluorescein sodium (0.1%; Tianjin Jingming, China) was applied to the corneal epithelium to observe the defects under cobalt blue light from a slit lamp (Optics Bridge, China). Corneal haze was observed and graded based on the haze grading method proposed by Zhou et al. (Zhou et al., 2020).

### Transmission Electron Microscopy

The mice were sacrificed and the eyeballs were fixed in 2.5% glutaraldehyde, dehydrated with ethanol and acetone, and embedded in epoxy resin. Ultrathin sagittal sections (60 nm) sliced using an Ultracut ultramicrotome (Ultracut, Leica) were placed on slot grids coated with polyvinyl formal, and counterstained with 1% uranium acetate.

### Histological and Immunohistochemical Staining

The eyeballs were fixed in 4% paraformaldehyde, dehydrated with a gradient of aqueous alcohol and xylene, and embedded in paraffin. The corneal structures were assessed by staining with hematoxylin-eosin (HE; Servicebio, China). Tissue sections were incubated with Ki-67 (12202, Cell Signaling Technology, United States) overnight. Antigens were retrieved; non-specific antigen binding was blocked; then the sections were incubated with the secondary antibody goat anti-rabbit IgG H&L (HRP; ab6721, Abcam) and 3, 3′-diaminobenzidine (Servicebio).

### TUNEL Staining

Label solution containing terminal deoxynucleotidyl transferase was incubated with paraffin-embedded sections in darkness for 2 h Nuclei were then stained with DAPI. Images were captured using a confocal microscope.

### Western Blotting

Corneas were suspended in lysis buffer (KeyGEN, China), homogenized (Jingxin, China), and centrifuged at 12,000 × g for 5 min at 4°C. Protein concentrations were determined using bicinchoninic acid standards (Leagene). Samples in loading buffer (Leagene), were denatured by boiling for 5 min, resolved by electrophoresis on 8–12% sodium dodecyl sulfate polyacrylamide gels (KeyGEN), then electroblotted onto polyvinylidene fluoride membranes (Millipore, United States). Non-specific antigen binding was blocked using 5% bovine serum albumin (Sigma Aldrich). The membranes were then incubated with primary antibodies Phospho-AMPKα (50081, Cell Signaling Technology), AMPKα (5831, Cell Signaling Technology), Phospho-mTOR (5536, Cell Signaling Technology), mTOR (2983, Cell Signaling Technology), Phospho-ULK1 (5869, Cell Signaling Technology), ULK1 (8054, Cell Signaling Technology), LC3B (2775, Cell Signaling Technology), SQSTM1/p62 (5114, Cell Signaling Technology), Beclin-1 (3495, Cell Signaling Technology), Bcl-2 (ab59348, Abcam), Bax (2772, Cell Signaling Technology), cleaved Caspase-3 (9664, Cell Signaling Technology) and β-Actin (AB0035, Abways Technology) for 16–20 h at 4 C. The membranes were washed, incubated with secondary antibody goat anti-rabbit IgG H&L (ab6721, Abcam), and examined using a chemiluminescence apparatus (LAS500, GE, United States). Images were analyzed using ImageJ.

### Real-Time Quantitative PCR

Total RNA was extracted using RNAiso Plus (9108, Takara, Japan) and quantified using a spectrophotometer (ND 2000c, Thermo Scientific). Gene expression was measured by real-time quantitative PCR (qPCR) using designed primers (Table 1), TB Green Premix (RR820A, Takara) and a real-time PCR system (CFX96, BioRad, United States).

**TABLE 1:** Sequence of primers for qPCR.

Gene	Species	Direction	Primer sequence 5'→3'
Cyclin A	Human	Forward	AACTTCAGCTTGTGGGCACT
		Reverse	CTGGTGGGTTGAGGAGAGAA
Cyclin E	Human	Forward	CCATCCTTCTCCACCAAAGA
		Reverse	AGCACCTTCCATAGCAGCAT
CDK2	Human	Forward	CCAGGAGTTACTTCTATGCCTGA
		Reverse	TTCATCCAGGGGAGGTACAAC
PCNA	Human	Forward	GCCAGAGCTCTTCCCTTACG
		Reverse	TAGCTGGTTTCGGCTTCAGG
GAPDH	Human	Forward	AAGAAGGTGGTGAAGCAGGC
		Reverse	TCCACCACCCTGTTGCTGTA
TNF- α	Mouse	Forward	ACCCTCACACTCAGATCATCTT
		Reverse	GGTTGTCTTTGAGATCCATGC
IL-1β	Mouse	Forward	TGCCACCTTTTGACAGTGATG
		Reverse	TGATGTGCTGCTGCGAGATT
IL-6	Mouse	Forward	TGATGGATGCTACCAAACTGGA
		Reverse	TGTGACTCCAGCTTATCTCTTGG
CXCL-2	Mouse	Forward	AAGGCAAGGCTAACTGACCTG
		Reverse	TTGGTTCTTCCGTTGAGGGAC
β-actin	Mouse	Forward	GATTACTGCTCTGGCTCCTAGC
		Reverse	GACTCATCGTACTCCTGCTTGC

### Statistical Analysis

Multiple comparisons were evaluated using the one-way analysis of variance (ANOVA) with Tukey or Dunnett’s post hoc tests using SPSS 24.0 (IBM, United States) and GraphPad Prism 9.0 (GraphPad Software, United States). Results are presented as the means ± standard deviation (SD) of three independent experiments, and differences were considered statistically significant at p < 0.05.

## Results

### Identification of hucMSCs and hucMSC-Exos

**FIGURE 2:**
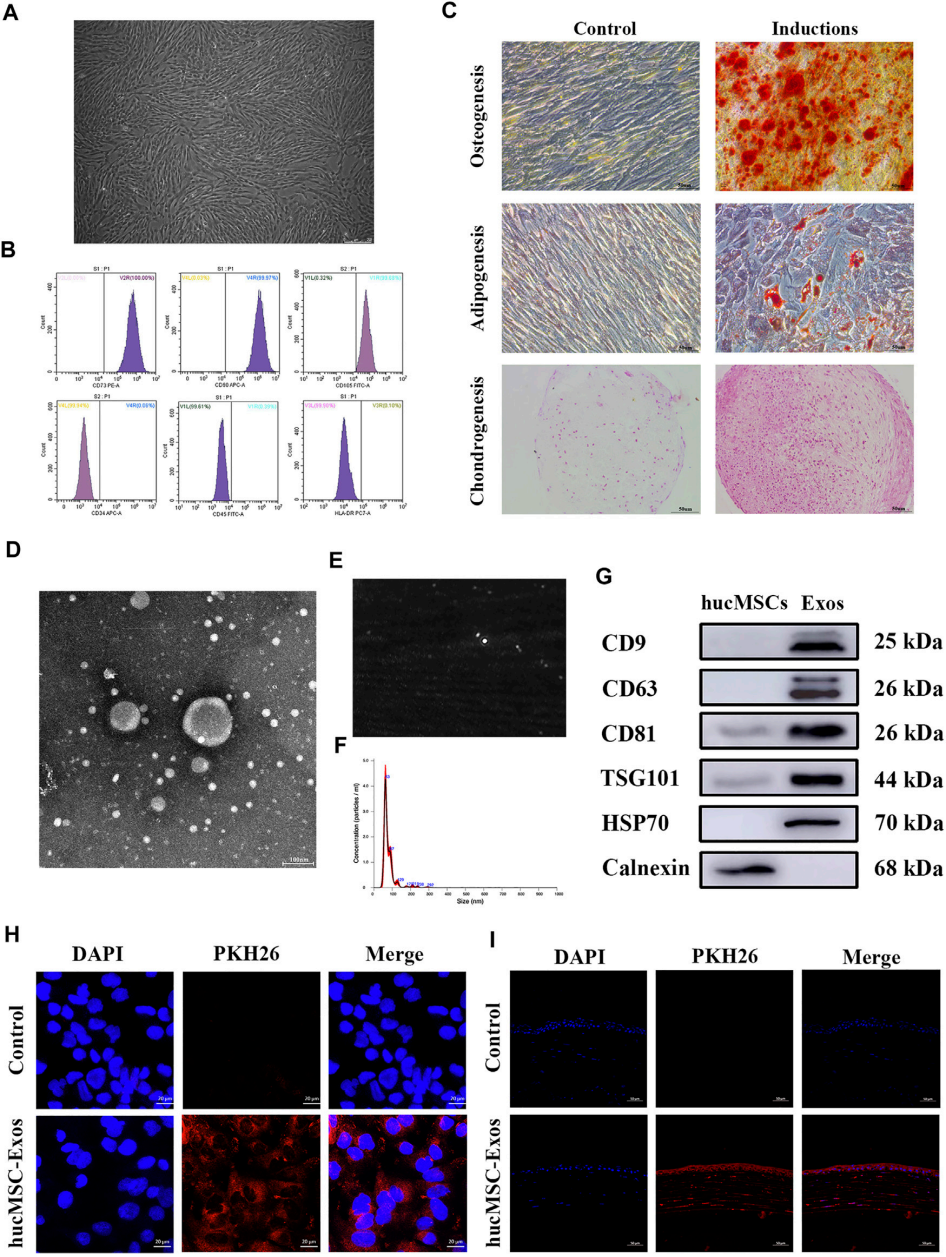
Identification of human umbilical cord mesenchymal stem cells (hucMSCs) and exosomes secreted from human umbilical cord mesenchymal stem cells (hucMSC-Exos). **(A)** Microscopic images of hucMSCs morphology. Scale bar: 250 μm. **(B)** Flow cytometry results for expression of stem cell surface markers CD73, CD90, CD105, CD34, CD45, and HLA-DR. **(C)** Alizarin red, Oil Red O, and Safranine O staining for osteogenic, adipogenic, and chondrogenic differentiation. Scale bar: 50 μm. **(D)** TEM images of hucMSC-Exos morphology. Scale bar: 100 nm. **(E,F)** NTA-determined size distribution of hucMSC-Exos. **(G)** Western blotting showing the expression of the markers (CD9, CD63, CD81, TSG101, HSP70, and Calnexin) of hucMSC-Exos. The lysate of hucMSCs served as the control group. **(H)** Uptake of hucMSC-Exos by HCECs *in vitro*. PBS was used as a negative control. Scale bar: 20 μm. **(I)** Uptake of hucMSC-Exos by cornea *in vivo*. Scale bar: 50 μm.

Typical spindle shapes were displayed by the hucMSCs (Figure 2A). Flow cytometry revealed that hucMSCs positively expressed the typical stem cell surface markers CD73, CD90, and CD105, but not the hematopoietic lineage markers CD34, CD45, and HLA-DR (Figure 2B), as previously reported by the International Society for Cellular Therapy (Dominici et al., 2006) A high degree of mineral deposits, intracytoplasmic lipid vacuoles and droplet accumulation, and positive chondrogenic mucopolysaccharide staining verified great potential for multi-directional differentiation (Figure 2C), confirming that the isolated cells were hucMSCs. Analysis of medium composition showed the exosomes did not exist in fresh DMEM/F12 (Supplementary Figure S1). HucMSC-Exos exhibited round or oval shapes with a bilayer membrane structure (Figure 2D), were distributed in a unimodal manner with an average diameter of 78.5 nm (Figures 2E,F), and positively expressed CD9, CD63, CD8, TSG101, and HSP70, while negatively expressed calnexin as previously reported (Théry et al., 2018b) (Figure 2G), suggesting successful extraction of high-purity exosomes. PKH26 is a lipophilic dye used to stain exosomes whose uptake can then be monitored. Red fluorescence was widely distributed in the cell cytoplasm (Figure 2H) and throughout the whole cornea at 24 h after the application of PKH26-labelled exosomes (Figure 2I). In contrast, no control cell showed intracellular red fluorescence either *in vivo* or *in vitro*.

### Effects of the Combination of hucMSC-Exos and Autophagy Regulators on HCECs

**FIGURE 3:**
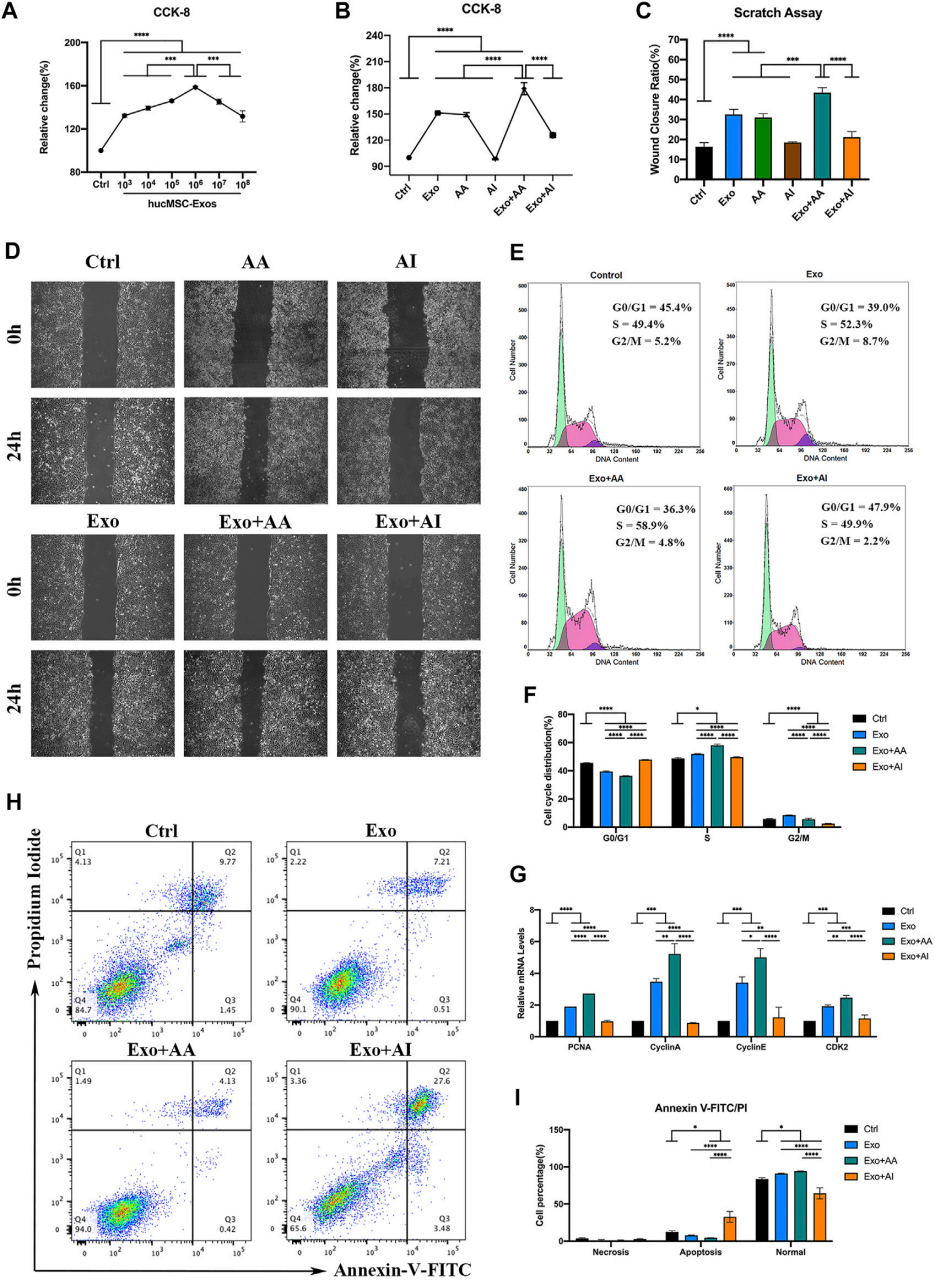
Effects of the combination of hucMSC-Exos and autophagy regulators on HCECs. **(A)** Cell-counting Kit-8 (CCK-8) assay for cell viability of HCECs incubated with different concentrations of hucMSC-Exos for 24 h **(B)** CCK-8 assay for cell viability of HCECs in Control, Exo, AA, AI, Exo + AA and Exo + AI groups for 24 h. **(C,D)** Scratch assay results for cell migration rates of HCECs in Control, Exo, AA, AI, Exo + AA and Exo + AI groups for 24 h. Scale bar: 250 μm. **(E,F)** Flow cytometry results for cell cycle composition of HCECs after combination treatment with hucMSC-Exos and autophagy regulators for 24 h. **(G)** Quantitative PCR results for mRNA expression of PCNA, Cyclin A, Cyclin E and CDK2 in HCECs. **(H, I)** Apoptosis assay results showing the proportion of HCECs at each phase after the treatment with hucMSC-Exos combined with autophagy regulators for 24 h. Data are shown as mean ± SD. *p < 0.05, **p < 0.01, ***p < 0.001, ****p < 0.0001. (Control: HCECs treated with PBS, Exo: HCECs treated with 1 × 10^6^/μl hucMSC-Exos, Exo + AA: HCECs treated with 1 × 10^6^/μl hucMSC-Exos and 50 nM Rapamycin, Exo + AI: HCECs treated with 1 × 10^6^/μl hucMSC-Exos and 5 μM Compound C).

Various concentrations of hucMSC-Exos increased cell viability within 24 h. Cell proliferation was particularly strong at 1.0 × 10^6^/μl (Figure 3A, p < 0.0001), which was determined as the optimal concentration (Exo). Compared with Exo, AA (50 nM Rapamycin) played similar role on accelerating cell viability and migration. In addition, Exo + AA (1 × 10^6^/μl hucMSC-Exos and 50 nM Rapamycin) showed increased cell viability compared with that shown by Exo and AA (Figure 3B, p < 0.001). Exo + AA significantly increased the wound closure areas (Figures 3C,D, Supplementary Table S1; p < 0.001). However, AI (5 μM Compound C) and Exo + AI (1 × 10^6^/μl hucMSC-Exos and 5 μM Compound C) showed little effect on above cell functions. The number of HCECs in the S phase, but a distinct decline in these parameters was observed during in the G0/G1 phase by cell cycle assay (Figures 3E,F, p < 0.0001). The promoted expression of proliferating cell nuclear antigen (PCNA), Cyclin A, Cyclin E, and Cyclin-dependent kinase 2 (CDK2) was detected in Exo + AA compared with that in other groups (Figure 3G, p < 0.05). Among the groups, the apoptosis percentage was the lowest in Exo + AA and the highest in Exo + AI (Figures 3H,I, Supplementary Table S2, p < 0.05).

### Autophagy and Inflammation in the Corneas of CI Mice

**FIGURE 4:**
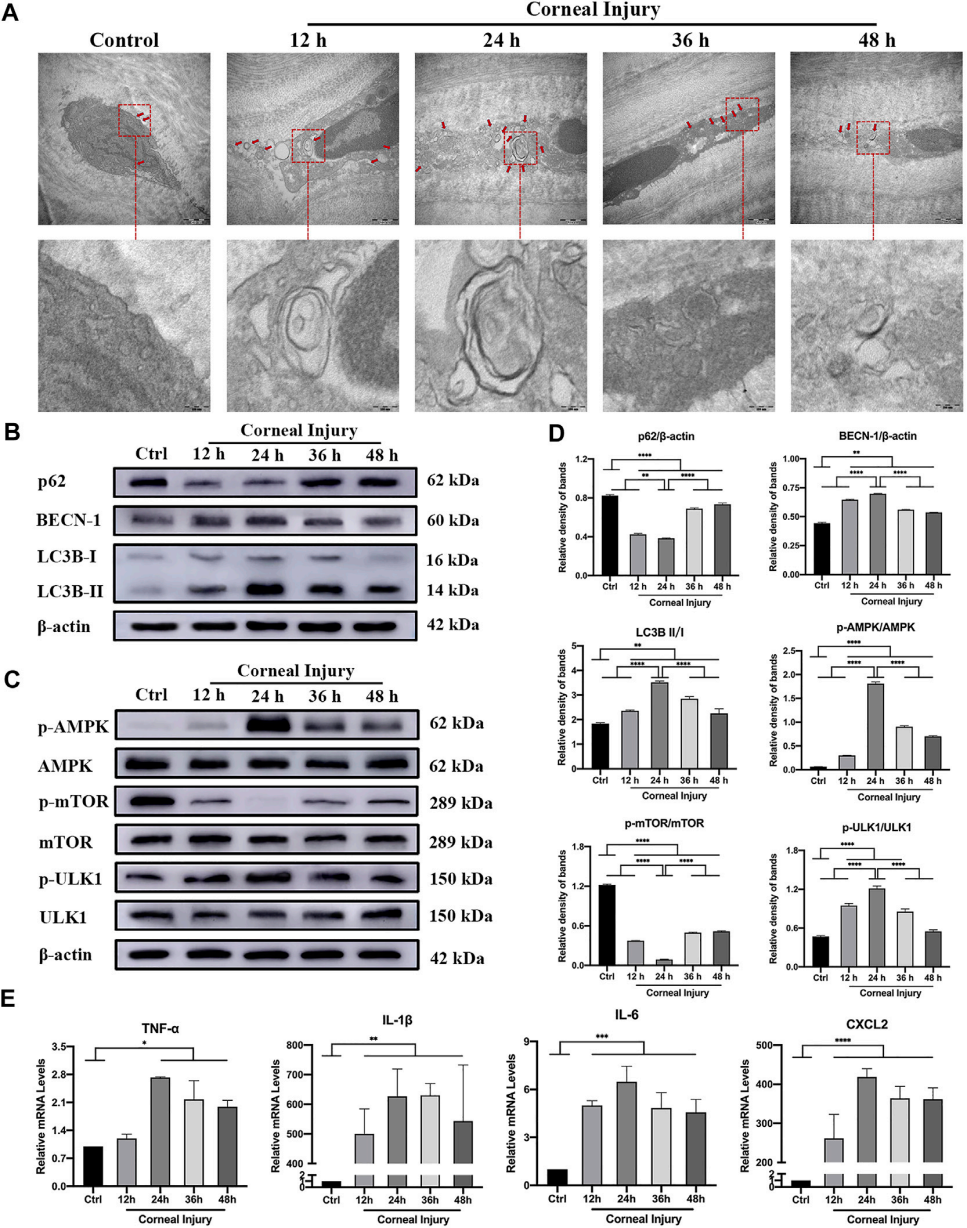
Autophagy and inflammation in the corneas of corneal injury (CI) mice. **(A)** Representative high magnification TEM pictures showing autophagosomes with double membrane structure were formed in CI models. Scale bar: 500 and 100 nm. **(B)** Western blotting showing expressions of the autophagy marker proteins p62, Beclin-1, and LC3B. **(C)** Western blotting showing the expression of the AMPK-mTOR-ULK1 autophagy flux pathway proteins pAMPK, AMPK, pULK1, ULK1, pmTOR and mTOR. **(D)** Relative band densities of p62, Beclin-1, LC3BII/I, and the ratios of pAMPK/AMPK, pULK1/ULK1, and pmTOR/mTOR. **(E)** Quantitative PCR results for mRNA levels of the inflammatory cytokines TNF-α, IL-1β, IL-6, and CXCL-2. Data are shown as mean ± SD. *p < 0.05, **p < 0.01, ***p < 0.001, ****p < 0.0001.

TEM showed an increased number of autophagosomes in higher magnification (Figure 4A) with double-layer membranes containing several protein metabolites, and severe intracellular organelle derangement showed that the expression of autophagic flux increased within 24 h and no longer continued to rise, suggesting autophagic activation. The increased expression of Beclin-1 and LC3B-II/I was accompanied by the degradation of the autophagy flux marker protein p62 in CI (Figure 4B). Western blotting showed that the phospho-AMPK/AMPK (pAMPK/AMPK) and phospho-ULK1/ULK1 (pULK1/ULK1) ratios significantly increased, and the phospho-mTOR/mTOR (pmTOR/mTOR) ratio decreased, especially at 24 h. These findings reflected autophagy activated by the AMPK-mTOR-ULK1 signaling pathway (Figures 4C,D; p < 0.05). In addition, the autophagy peaked at 24 h, then reached a plateau. The mRNA expression of proinflammatory cytokines measured using qPCR showed that tumor necrosis factor alpha (TNF-α), interleukin-1 beta (IL-1β), interleukin-6 (IL-6) and C-X-C ligand-2 (CXCL-2) expression were significantly elevated within 24 h, which then remained above baseline levels (Figure 4E, p < 0.05).

### Effects of hucMSC-Exos on CI Mouse Corneas

**FIGURE 5:**
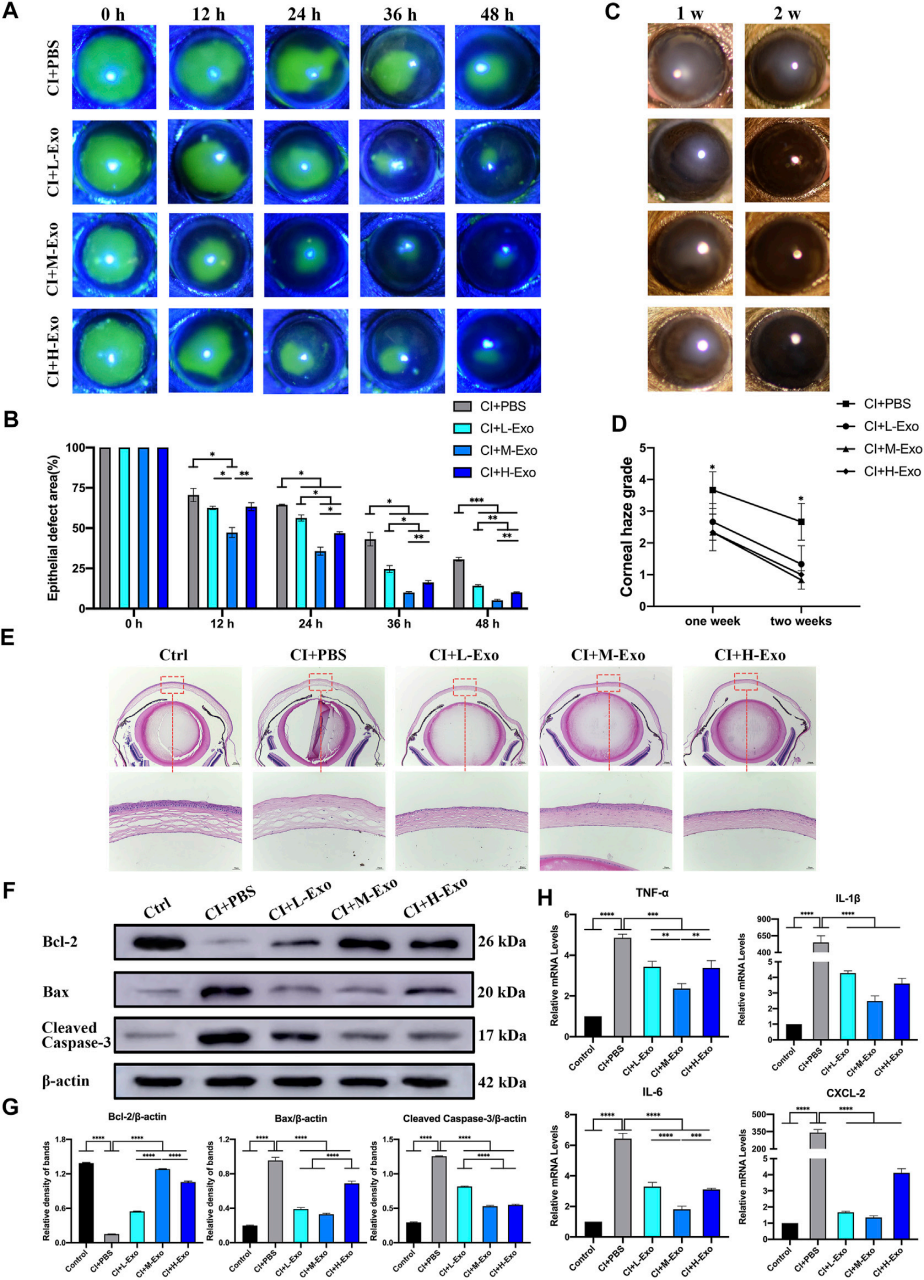
Effect of hucMSC-Exos on CI mouse corneas. **(A,B)** Cornea fluorescein staining; representative images of the corneal epithelial defect areas. Data are quantified in the bar graph. **(C,D)** Slit lamp showing the representative images of corneal haze. Haze grading data are quantified in line chart. **(E)** Mouse eyeballs were harvested at 48 h post-injury. HE staining for histologic structure of cornea wound healing. Scale bar: 250 and 50 μm. **(F,G)** Western blotting showing the apoptosis related proteins Bcl-2, Bax, and cleaved Caspase-3. Relative band densities are quantified in bar graphs. **(H)** Quantitative PCR results for mRNA levels of the inflammatory cytokines TNF-α, IL-1β, IL-6, and CXCL-2, and quantified in bar graphs. Data are shown as mean ± SD. *p < 0.05, **p < 0.01, ***p < 0.001, ****p < 0.0001. (Control: normal corneas treated with PBS, CI + PBS: injured corneas treated with PBS, CI + L-Exo: injured corneas treated with 1 × 10^5^/μl hucMSC-Exos, CI + M-Exo: injured corneas treated with 1 × 10^6^/μl hucMSC-Exos, CI + H-Exo: injured corneas treated with 1 × 10^7^/μl hucMSC-Exos).

The recovery of residual epithelial defect areas in injured mouse corneas was accelerated (Figures 5A,B, Supplementary Table S3, p < 0.05), and haze grades were considerably reduced in the CI + L-Exo, CI + M-Exo, and CI + H-Exo groups compared with those in the CI + PBS group (Figures 5C,D, p < 0.05). Hematoxylin-eosin (HE) staining showed only 0–1 layer of corneal epithelial cells in the central cornea with disordered arrangement of stromal fibers in the CI + PBS group, almost two to three integrated layers of cells in the CI + L-Exo and CI + H-Exo groups, and three to five layers in the CI + M-Exo group (Figure 5E). Inflammatory cell infiltration was significantly ameliorated, and the structures were more ordered in the Exo treated groups. Levels of the apoptosis proteins Bcl-2-associated X (Bax), cleaved Caspase-3, and the remarkably downregulated anti-apoptosis protein B-cell lymphoma-2 (Bcl-2) were significantly downregulated in CI + PBS. However, hucMSC-Exos reversed these trends to various degrees (Figures 5F,G, p < 0.05). The hucMSC-Exos appeared to decrease the levels of the inflammation-associated factors TNF-α, IL-1β, IL-6, and CXCL-2 compared with the CI + PBS group; and CI + M-Exo significantly decreased those of the levels TNF-α and IL-6 compared with the CI + L-Exo and CI + H-Exo groups (Figure 5H, p < 0.01).

### Effects of hucMSC-Exos Combined With Autophagy Regulators on Corneal Clinical and Pathological Presentation

**FIGURE 6:**
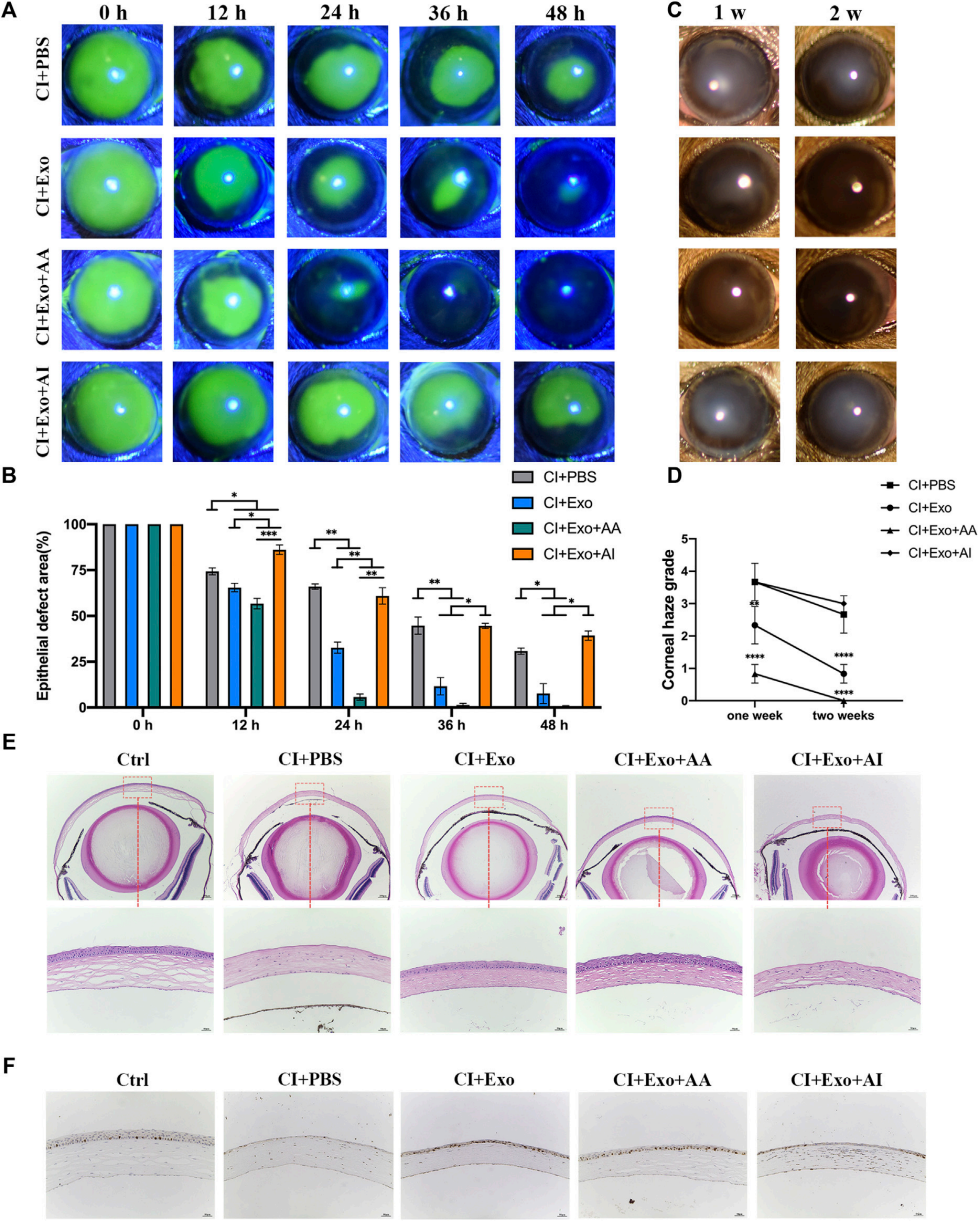
Effect of combination treatment with hucMSC-Exos and autophagy regulators on corneal clinical and pathological examination. **(A,B)** Corneal fluorescein staining showing the representative images of the corneal epithelial defect areas. Data are quantified in the bar graph. **(C,D)** Slit lamp showed the representative images of corneal haze. Haze-grading data were quantified using a line chart. **(E)** Mouse eyeballs were harvested at 48 h post-injury. HE staining for the histological structure of cornea wound healing. Scale bar: 250 and 50 μm. **(F)** Mouse eyeballs were harvested at 48 h post-injury. Immunohistochemical staining showing expression of the proliferation marker Ki-67. Data are shown as mean ± SD. *p < 0.05, **p < 0.01, ***p < 0.001, ****p < 0.0001. (Control: normal corneas treated with PBS, CI + PBS: injured corneas treated with PBS, CI + Exo: injured corneas treated with 1 × 10^6^/μl hucMSC-Exos, CI + Exo + AA: injured corneas treated with 1 × 10^6^/μl hucMSC-Exos and 10 μM Rapamycin, CI + Exo + AI: injured corneas treated with 1 × 10^6^/μl hucMSC-Exos and 50 μM Compound C).

Representative images showed that the fluorescence staining scores for the cornea under the slit lamp were significantly low in CI + Exo + AA, but remained high in the CI + Exo + AI and did not differ from those of CI + PBS (Figures 6A,B, Supplementary Table S4, p < 0.05). Similarly, the haze grade of the CI + Exo + AA group was markedly reduced compared with other groups (Figures 6C,D, p < 0.0001). Corneal regeneration was also promoted by CI + Exo + AA as evidenced by the more integrated and ordered layers of the corneal epithelium (Figure 6E). Moreover, the expression of proliferation protein Ki-67 was elevated in the CI + Exo + AA group compared that in other groups (Figure 6F).

### Effects of hucMSC-Exos Combined With Autophagy Regulators on Autophagy Levels

**FIGURE 7:**
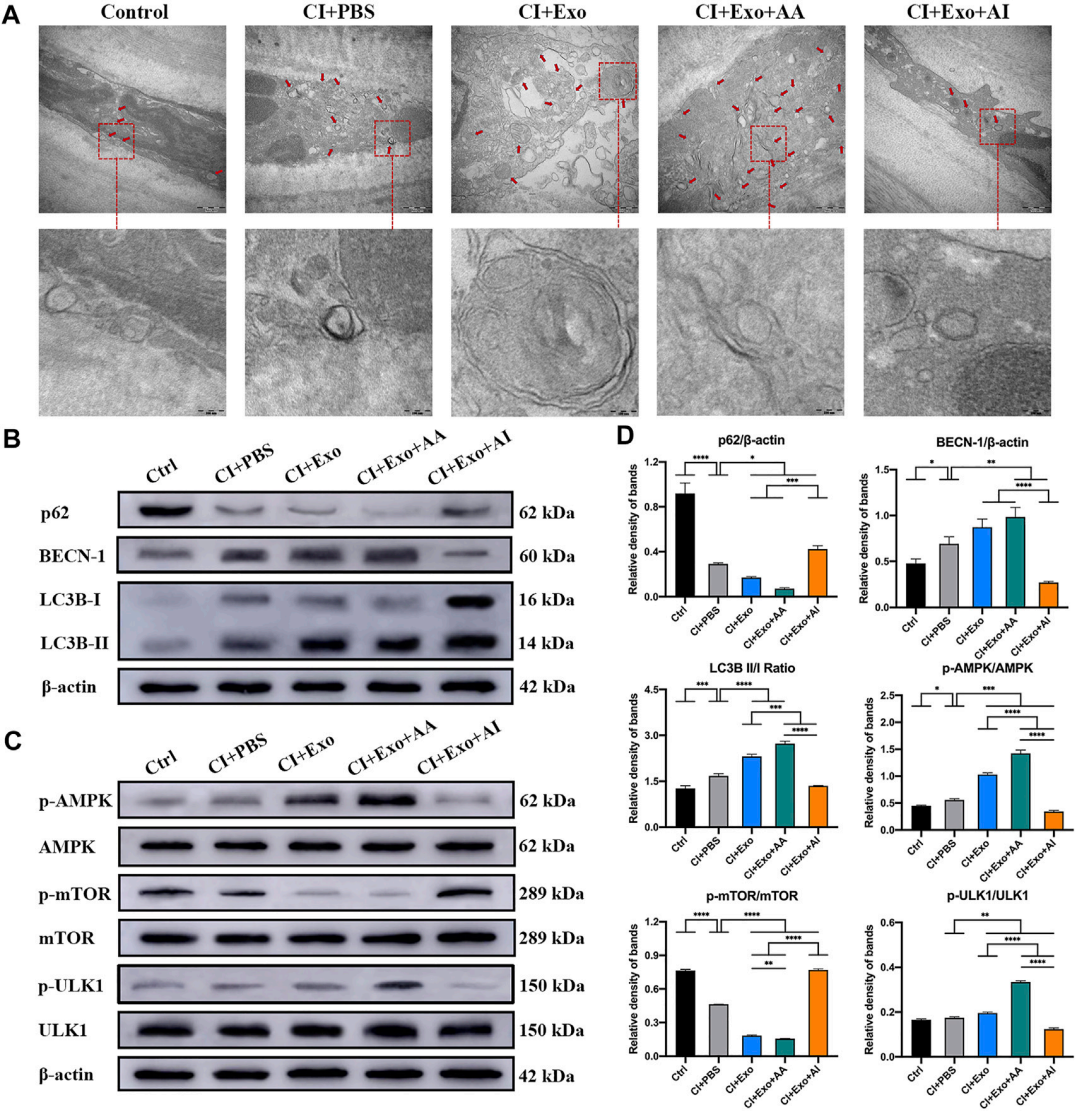
Effect of combination treatment with hucMSC-Exos and autophagy regulators on autophagy levels in CI mouse corneas. **(A)** Representative high-magnification TEM images showing the autophagosome counts in the groups. Scale bar: 500 and 100 nm. **(B)** Western blotting showing autophagy markers p62, Beclin-1, and LC3B. **(C)** Western blotting showing the AMPK-mTOR-ULK1 autophagy flux pathway proteins pAMPK, AMPK, pULK1, ULK1, pmTOR and mTOR. **(D)** Relative band densities of p62, Beclin-1, LC3B II/I, pAMPK/AMPK, pULK1/ULK1, and pmTOR/mTOR, quantified in bar graphs. Data are shown as mean ± SD. *p < 0.05, **p < 0.01, ***p < 0.001, ****p < 0.0001. (Control: normal corneas treated with PBS, CI + PBS: injured corneas treated with PBS, CI + Exo: injured corneas treated with 1 × 10^6^/μL hucMSC-Exos, CI + Exo + AA: injured corneas treated with 1 × 10^6^/μL hucMSC-Exos and 10 μM Rapamycin, CI + Exo + AI: injured corneas treated with 1 × 10^6^/μl hucMSC-Exos and 50 μM Compound C).

Compared with the CI + PBS group, the CI + Exo + AA group had normal cells with compact organelles or enlarged autophagosomes, including degraded organelles, and an increasing number of autophagic vesicles with a multilayer membrane structure. However, the CI + Exo + AI group had fewer vacuole-like structures, endoplasmic reticulum dilation, and ribosome abscission (Figure 7A). The expression of Beclin-1 and LC3B-II/I was enhanced, but p62 expression was reduced by Rapamycin. In contrast, the expression of Beclin-1 and LC3B-II/I was reduced, whereas that of p62 was enhanced by Compound C (Figure 7B). Meanwhile, CI + Exo + AA significantly increased the pAMPK/AMPK and pULK1/ULK1 ratios and decreased the pmTOR/mTOR ratio, while CI + Exo + AI exhibited the opposite trend (Figures 7C,D, p < 0.05).

### Effects of hucMSC-Exos Combined With Autophagy Regulators on Apoptosis and Inflammation Levels in CI Mice

**FIGURE 8:**
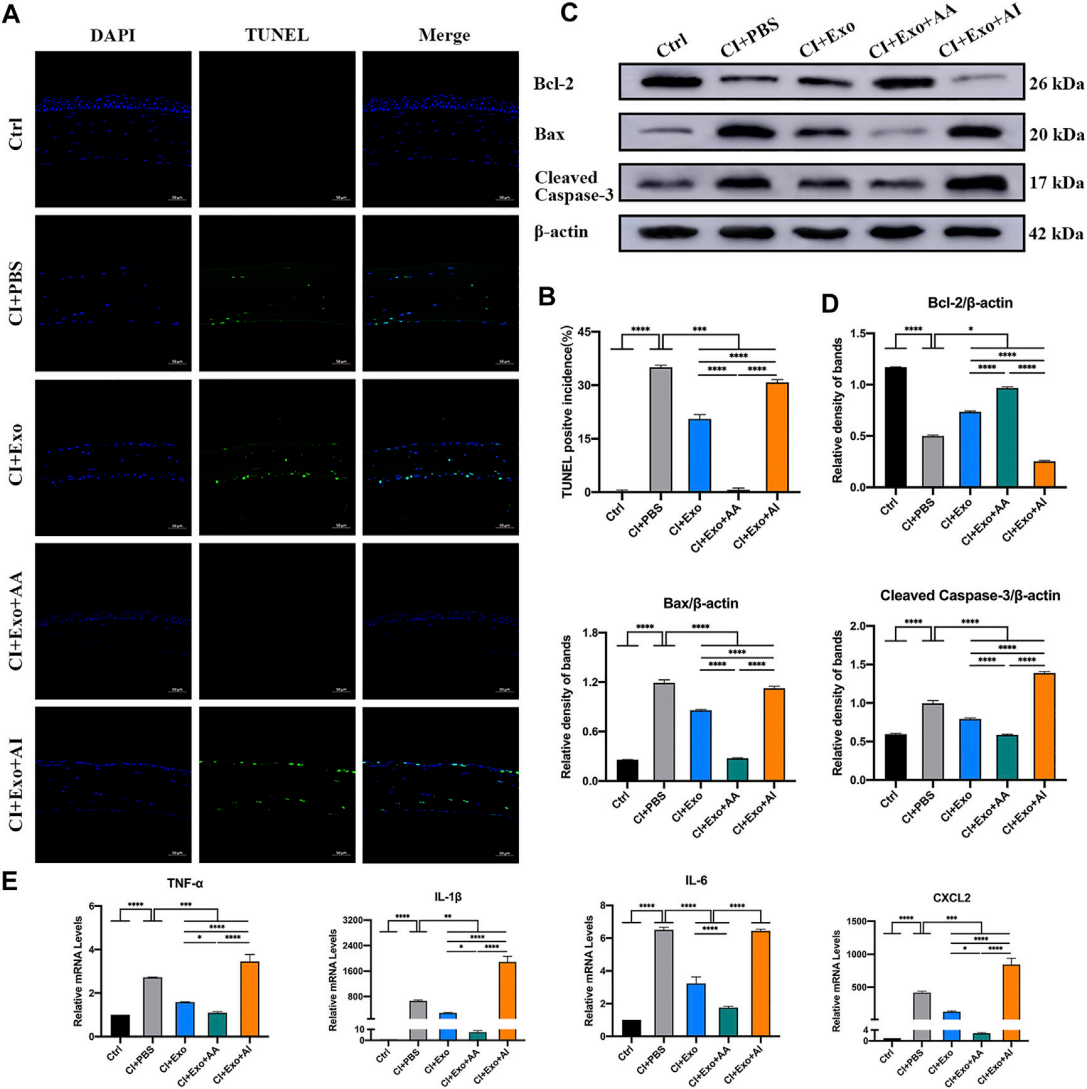
Effect of combination treatment with hucMSC-Exos and autophagy regulators on apoptosis and inflammation in CI mice. **(A,B)** Mouse eyeballs were harvested at 48 h post-injury. Representative TUNEL staining images of apoptotic cells. Positive incidences are shown in the bar graph. Scale bar: 50 μm. **(C,D)** Western blotting showing the apoptosis-related proteins Bcl-2, Bax, and cleaved Caspase-3. The relative band densities were quantified using bar graphs. **(E)** Quantitative PCR results for mRNA levels of the pro-inflammatory factors TNF-α, IL-1β, IL-6, and CXCL-2, and quantified in bar graphs. Data are shown as mean ± SD. *p < 0.05, **p < 0.01, ***p < 0.001, ****p < 0.0001. (Control: normal corneas treated with PBS, CI + PBS: injured corneas treated with PBS, CI + Exo: injured corneas treated with 1 × 10^6^/μl hucMSC-Exos, CI + Exo + AA: injured corneas treated with 1 × 10^6^/μl hucMSC-Exos and 10 μM Rapamycin, CI + Exo + AI: injured corneas treated with 1 × 10^6^/μl hucMSC-Exos and 50 μM Compound C).

The CI + Exo + AA corneas contained more live, ordered, and non-apoptotic cells within 48 h of treatment compared with the CI + PBS, CI + Exo, and CI + Exo + AI groups. The apoptosis percentage was slightly reduced in CI + Exo + AI, but higher than that in the Exo group (Figures 8A,B, p < 0.001). In the CI + Exo + AA group, the expression levels of Bax and cleaved Caspase-3 were decreased and Bcl-2 was increased compared with other groups. In contrast, the expression trends in the CI + Exo + AI group were the opposite (Figures 8C,D, p < 0.05). The expressions of TNF-α, IL-1β, IL-6, and CXCL-2 were distinctly down-regulated in the CI + Exo + AA compared with the CI + PBS group. However, high levels of TNF-α, IL-1β, and CXCL-2 were expressed in the CI + Exo + AI (Figure 8E, p < 0.05).

## Discussion

Rapid and complete wound closure promotes higher transparency and better preservation of vision during corneal wound healing. To date, studies on CI treatment involving hucMSC-Exos and autophagy regulators are still limited, and no previous study has explored the mechanism by which these combined treatments affect corneal wound healing. Thus, we focused on exploring the molecular mechanisms by which hucMSC-Exos affect autophagy in HCECs and a CI model. Our findings suggested that hucMSC-Exos combined with an autophagy activator contribute remarkably to enhancing HCEC functions and alleviating corneal defects, apoptosis and inflammation by activating the AMPK-mTOR-ULK1 pathway that is associated with autophagy flux. They also provide a new therapeutic approach to corneal wound healing and other ocular surface diseases.

Firstly, the isolated cells positively expressed CD73, CD90, and CD105, but negatively expressed CD34, CD45, and HLA-DR, confirming that the isolated cells were indeed hucMSCs. Typical bilayer morphology and size with expression of CD9, CD63, CD8, TSG101, and HSP70 suggested successful extraction of high-purity exosomes. PKH26-labelled exosomes distributed in the cell cytoplasm and throughout the cornea indicated that hucMSC-Exos were successfully fused and taken up by HCECs and the corneas.

In the *in vitro* experiments, we found the effects of various exosome concentrations on HCEC proliferation were promoted, which was similar to that found by Wang et al. (Wang et al., 2020b). As previous studies showed (Ma et al., 2020; Chen, 2021), different contents of extracellular vesicles result in different effects, which positively or negatively regulate cell proliferation and migration ability (Eirin et al., 2017). We identified the optimal concentration (1.0 × 10^6^/μl) and applied to the following tests as the Exo group. Then, we systematically investigated the effects of hucMSC-Exos, autophagy activator, autophagy inhibitors, and hucMSC-Exos combined with those autophagy regulators on HCECs. Exosomes and Rapmycin both promoted cell proliferation and migration, but there was no significant difference between them. Interestingly, the combination treatment of hucMSC-Exos and Rapmycin could further contribute those positive effects. The optimal mechanism of tissue repair is that remaining live cells reenter the cell cycle (Wang et al., 2020a). The proportions of cells in the S phase and in the G0 to G1 phase increased and decreased, respectively, when incubated with the exosomes combined with an autophagy activator. Therefore, hucMSC-Exos with the autophagy activator promoted cell cycle progression. The content of PCNA in proliferating cells is periodic, and PCNA expression from the late G1 to mid S phases reaches a peak that can reflect the activity of DNA replication (Li T. et al., 2019). Taking the example of Cyclins and their catalytic partner CDKs, the G1 to G2 phase transition is promoted by Cyclin E-CDK2 and Cyclin A-CDK2 complexes (Chandrasekher et al., 2014). Our results showed that hucMSC-Exos combined with an autophagy activator helped to promote the HCEC cell cycle by upregulating PCNA, Cyclin A, Cyclin E, and CDK2. Similarly, flow cytometry revealed that the apoptotic rate of HCECs was reduced after incubation with hucMSC-Exos with or without an autophagy activator. Moreover, the combination exerted more powerful effects on these functions. Therefore, we concluded that hucMSC-Exos combined with an autophagy activator promote cell proliferation, migration capacity, cycle progression, and apoptosis inhibition. When combined with an autophagy inhibitor, these cell functions were not promoted compared with treatment with PBS. Since autophagy activators can dramatically enhance cell functions, the possibility of hucMSC-Exos affecting autophagy was the focus of our subsequent animal experiments.

For the *in vivo* experiments, we established and monitored a murine model with mechanical CI for 48 h. Our findings showed for the first time that the formation of numerous autophagosomes, elevated autophagic marker protein expression in the mechanically injured corneas. The occurrence and development of autophagy uncovered herein suggested that autophagy is directly related to corneal homeostasis. The level of autophagy is low in healthy corneas, and autophagic activation through the AMPK-mTOR-ULK1 signaling pathway was associated with mechanical ocular surface damage. Autophagy induction and the release of numerous pro-inflammatory cytokines were most likely owing to CI-induced stress responses (Levine et al., 2011). In the event of cell death, the release of cytokines, such as TNF-α, IL-1β, IL-6, and CXCL-2 from injured epithelium promotes neutrophil infiltration towards damaged tissues resulting in exacerbated inflammation (Ljubimov and Saghizadeh, 2015). Our data were consistent with those conclusions. Meanwhile, although we found elevated autophagy levels in corneas and a reduced area of defective corneal epithelium within 24 h after injury, inflammation on the ocular surface was not relieved. Thereafter, the autophagy level decreased, but the expression of TNF-α, IL-1β, IL-6, and CXCL-2 levels remained high. This indicated that the increased levels of autophagy in response to CI-related stress are transient and limited. This change of autophagic level was similar in dry eye which was found in our previous study (Ma et al., 2019). This indicated that the cornea could not easily remove all impaired organelles and cytoplasmic contents to maintain a steady state via finite lysosomal pathways. Based on previous findings suggesting that autophagy is involved in regulating the ocular balance (Feng et al., 2021), we speculate that the rate of CI healing is related to the expression level of autophagy. A suitable autophagic level is therefore conducive to the treatment and prognosis.

**FIGURE 9:**
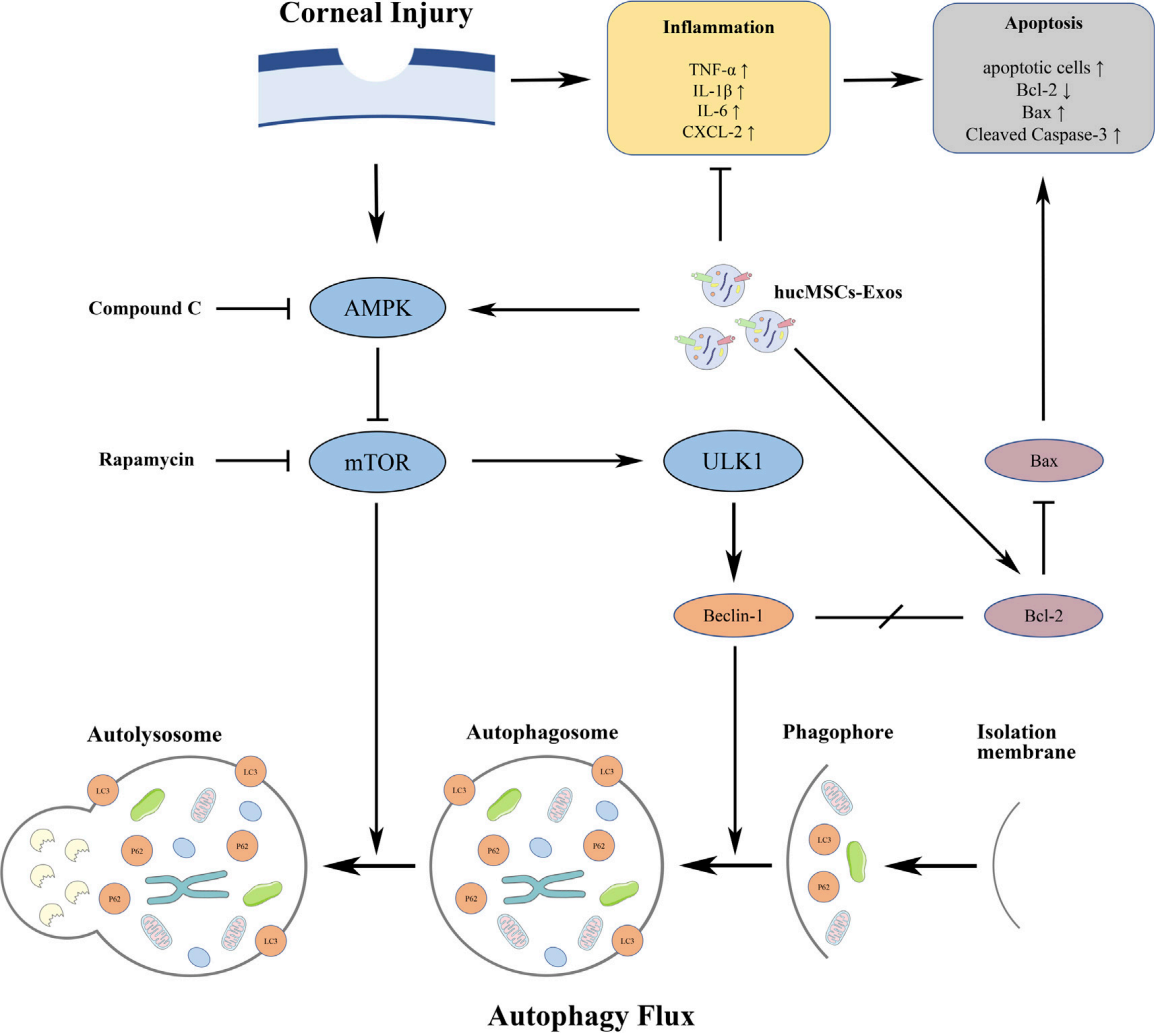
Diagram of the proposed mechanisms underlying the functions of hucMSC-Exos and hucMSC-Exos combined with autophagy regulators on corneal injury.

Next, we compared the effects of various concentrations of hucMSC-Exos and combined them with autophagy regulators to treat corneal defects. The results showed that hucMSC-Exos exerted therapeutic effects, and that the optimal concentration for corneal epithelial defect healing *in vivo* was 1.0 × 10^6^/μl, which was similar to the results *in vitro*. In addition to accelerating corneal re-epithelialization and reducing haze grades, exosomes exerted anti-apoptotic and anti-inflammatory effects on corneal wound healing. These were significantly reflected in the decreased levels of apoptotic cells, Bax, cleaved Caspase-3, and TNF-α, IL-1β, IL-6, and CXCL-2, and the increased level of Bcl-2. The mechanisms of apoptosis, inflammation and autophagy are interrelated. Physical wounds trigger the release of inflammatory cytokines from epithelial cells and the rapid apoptosis of keratocytes (Maycock and Marshall, 2014). AMPK-stimulated autophagy dissociates the Beclin1-Bcl-2 complex to inhibit apoptosis (Maiuri et al., 2007; He et al., 2013) and works on the growth, development, and homeostasis of inflammatory cells, that are conducive to recovery from apoptotic and inflammatory diseases (Morgan-Bathke et al., 2013; Rufini et al., 2015; Zhang et al., 2016). In this study, the AMPK-mTOR-ULK1 autophagy flux pathway was effectively activated or inhibited by hucMSC-Exos combined with an autophagy activator or inhibitor under the CI conditions. The combination of hucMSC-Exos with the autophagy activator Rapamycin exerted similar therapeutic effects to appropriate concentrations of exosomes. Furthermore, the recovery speed of corneal reconstruction as well as the anti-apoptotic and anti-inflammatory effects were improved compared with those under exosome treatment alone. Based on these findings, hucMSC-Exos combined with autophagy activators should be further explored as an important part of regenerative medicine for corneal wound healing. Contrary to the promising effects of treatment with hucMSC-Exos alone or combined with autophagy activators, combination with the autophagy inhibitor Compound C had little effect on corneal epithelial and stromal healing. The expression of apoptotic and inflammatory factors in the autophagy inhibitor groups was equivalent to, or exceeded those with the treatment of PBS. The proposed mechanisms underlying the roles of hucMSC-Exos and hucMSC-Exos combined with autophagy regulators on corneal injury are shown in Figure 9. The underlying association of exosomes and autophagy during the pathogenesis and treatment of CI requires further investigations. Additionally, we plan to further investigate different autophagy modulators and optimal concentrations to form a more complete body of useful information.

## Conclusion

Our findings demonstrated that autophagy plays an essential role in corneal wound healing. HucMSC-Exos, when combined with autophagy activators, exerted protective effects on mechanical CI and accelerated cell proliferation and migration whilst attenuating apoptosis and inflammation via the activation of the AMPK-mTOR-ULK1 autophagy pathway. Therefore, exosomes and autophagy activators may represent a novel approach to corneal wound healing and ocular surface regeneration.

## Data Availability

The original contributions presented in the study are included in the article/Supplementary Material, further inquiries can be directed to the corresponding author.
