# Supplementary material for: Exosomes From Human Umbilical Cord Mesenchymal Stem Cells Treat Corneal Injury via Autophagy Activation
Source: Front Bioeng Biotechnol. 2022 Apr 11;10:879192. doi: 10.3389/fbioe.2022.879192 (PMC9063640; doi:10.3389/fbioe.2022.879192)
Supplement: Supplementary file 1 [file DataSheet1.docx]

Supplementary Material

# Supplementary Data

## Supplementary Figures


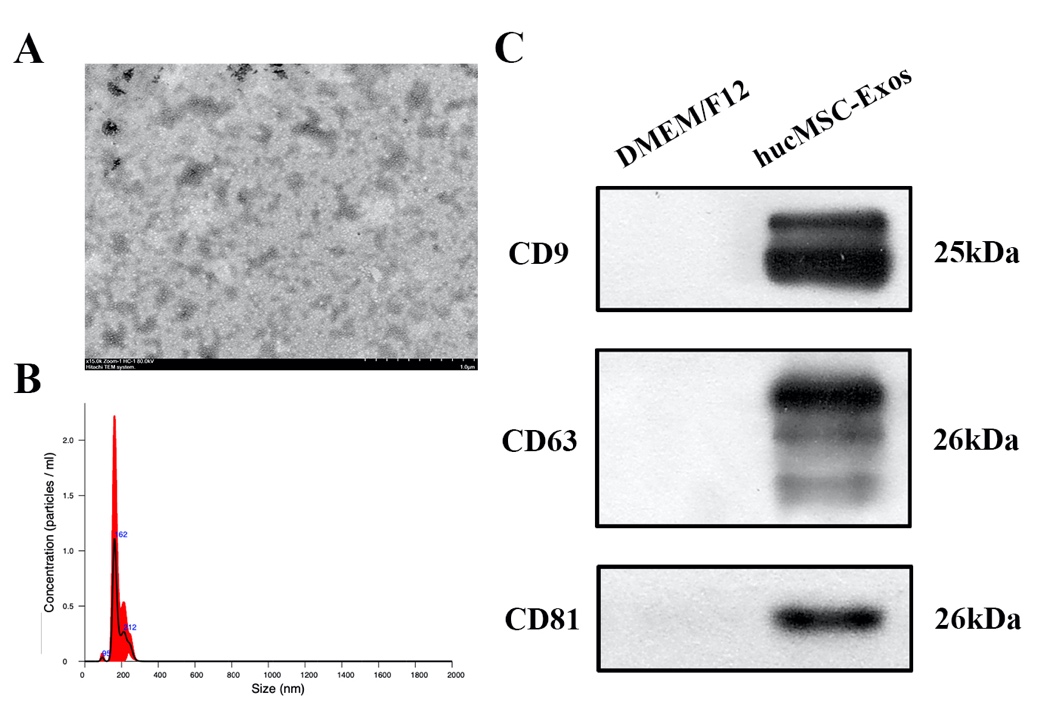


**Supplementary Figure 1.** (**A**) TEM images of DMEM/F12. Scale bar: 1 μm. (**B**) NTA-determined size distribution of DMEM/F12. (**C**) Western blot images for expression of the markers (CD9, CD63, CD81) of DMEM/F12. The hucMSC-Exos served as the control group.

## Supplementary Tables

| Groups | Wound Closure Areas |
| --- | --- |
| Ctrl | 16.36 ± 2.109% |
| Exo  AA  AI | 32.51 ± 2.568%  31.01 ± 1.894%  18.50 ± 0.368% |
| Exo+AA | 43.38 ± 2.578% |
| Exo+AI | 21.16 ± 2.823% |

**Supplementary Table 1.** Eﬀect of the combination of hucMSCs-Exos and autophagy regulator on the scratch assay of HCECs.

| Groups | The Proportion of Apoptotic Cells |
| --- | --- |
| Ctrl | 12.487 ± 1.445% |
| Exo | 7.983 ± 0.327% |
| Exo+AA | 4.540 ± 0.272% |
| Exo+AI | 32.783 ± 7.364% |

**Supplementary Table 2.** Eﬀect of the combination of hucMSCs-Exos and autophagy regulator on the cell apoptosis assay of HCECs.

| Groups | 12 h | 24 h | 36 h | 48 h |
| --- | --- | --- | --- | --- |
| CI+PBS | 70.597 ± 4.067% | 64.461 ± 0.349% | 43.138 ± 4.199% | 30.608 ± 1.203% |
| CI+L-Exo | 62.574 ± 1.012% | 56.287 ± 1.911% | 24.614 ± 2.162% | 14.245 ± 0.605% |
| CI+M-Exo | 47.272 ± 3.125% | 35.738 ± 2.451% | 9.952 ± 0.601% | 5.148 ± 0.557% |
| CI+H-Exo | 63.309 ± 2.411% | 46.803 ± 0.919% | 16.334±1.120% | 10.024 ± 0.371% |

**Supplementary Table 3.** Eﬀect of hucMSCs-Exos on the residual corneal epithelial defect areas.

| Groups | 12 h | 24 h | 36 h | 48 h |
| --- | --- | --- | --- | --- |
| CI+PBS | 74.258 ± 1.914% | 65.983 ± 1.460% | 44.743 ± 4.666% | 30.763 ± 1.697% |
| CI+Exo | 65.492 ± 2.261% | 32.651 ± 3.054% | 11.652 ± 4.745% | 7.595 ± 5.434% |
| CI+Exo+AA | 56.708 ± 2.818% | 5.713 ± 1.679% | 1.358 ± 0.923% | 0.379 ± 0.656% |
| CI+Exo+AI | 86.098 ± 2.575% | 60.912 ± 4.428% | 44.518 ± 1.399% | 39.255 ± 2.563% |

**Supplementary Table 4.** Eﬀect of the combination of hucMSCs-Exos and autophagy regulators on the residual corneal epithelial defect areas.
